# Pulp-to-Pulp Pinch Reconstruction in a Tetraplegic Patient Utilizing Nerve and Tendon Transfers: A Case Report

**DOI:** 10.7759/cureus.43755

**Published:** 2023-08-19

**Authors:** Jayme A Bertelli, Vera L Lehm

**Affiliations:** 1 Surgery, Federal University of Santa Catarina, Florianopolis, BRA; 2 Orthopedics and Traumatology, Governador Celso Ramos Hospital, Florianopolis, BRA; 3 Hand Therapy, Vera Lehm Hand Clinic, Joinville, BRA

**Keywords:** nerve repair, tendon transfer, nerve transfer, spinal cord injury, tetraplegia

## Abstract

In tetraplegia, hand reconstruction is of high priority. Key pinch reconstruction has been advocated for tetraplegia hand reconstruction because of the lack of donors for nerve and tendon transfers. We report a patient with mid-cervical tetraplegia who underwent nerve and tendon transfers in the right and left upper limbs seven months post-injury to reconstruct hand function. The particularity of our case resides in the left-hand thumb and index pulp-to-pulp reconstruction. For this, we transferred the nerve to the supinator to the posterior interosseous nerve and the nerve to the extensor carpi radialis brevis to the anterior interosseous nerve. During a second surgery, we relieved clawing by transferring the split flexor digitorum superficialis of the middle and ring fingers, motored by the brachioradialis, to the extensor apparatus of all fingers. Finally, to achieve better thumb palmar abduction, we osteotomized the scaphoid tubercle and fixed it to the distal radius while maintaining thenar muscle attachments. Five years after surgery, the patient was able to grasp and release small objects placed on a table after becoming left-handed. Here, we demonstrated that pinch-to-pinch reconstruction is possible, which increased hand use in daily activities, especially during eating and grabbing small objects over the table.

## Introduction

In tetraplegic patients with strong wrist extensors, elbow extension and finger flexion can be reconstructed using nerve or tendon transfers. However, finger and thumb active extensions are reconstructed adequately only with nerve transfers [1].

If wrist extension is strong and pronator teres is active, the extensor carpi radialis longus (ECRL), extensor carpi radialis brevis (ECRB), and the supinator muscle are preserved. The nerves to the ECRB and supinator are excellent donors for finger flexion and extension reconstruction, respectively [2].

With tetraplegia, because of limited donor availability, only key pinch reconstruction has been advocated [3,4]. However, the key pinch does not allow patients to grasp small objects placed on a flat surface. This task needs the thumb and index pulp to come together.

We report the clinical case of a patient with tetraplegia who underwent pulp-to-pulp reconstruction employing nerve and tendon procedures to restore elbow extension, finger flexion/extension, and thumb abduction while relieving hand clawing.

## Case presentation

After falling off a roof, a 48-year-old male sustained a complete cervical spinal cord injury. Seven months after his accident, in his right upper limb, elbow extension was impaired, wrist extension was weak (4.8 kg), and finger flexion and extension were paralyzed. On the left side, elbow extension was preserved, scoring M4. Wrist extension was strong (9.8 kg). Both the ECRL and ECRB were working. The flexor carpi radialis and pronator teres were preserved. According to the American Spinal Injury Association/International Spinal Cord Society (ASIA/ISCoS) International Standards for Neurological Classification of Spinal Cord Injury [5], our patient was classified as having C6 spinal-level paralysis.

**Figure 1:**
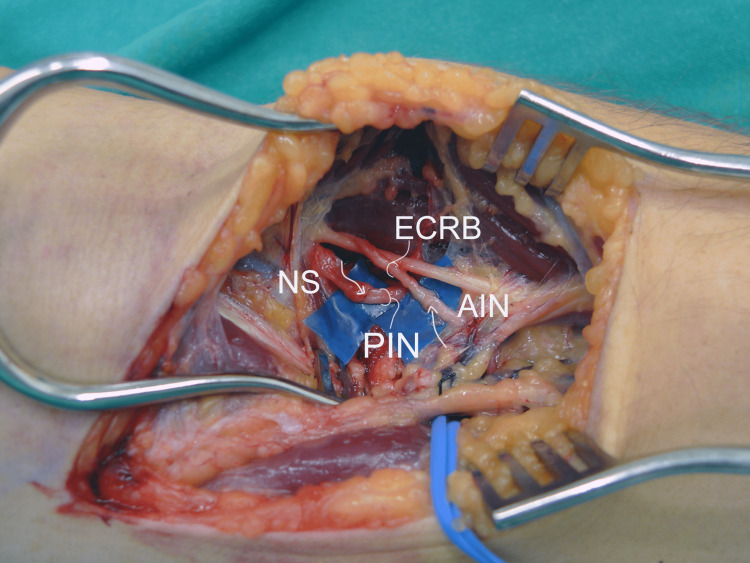
Intraoperative view of the anterior aspect of the left forearm depicting the transfer of the nerve to supinator (NS) to the posterior interosseous nerve (PIN) and of the extensor carpi radialis brevis (ECRB) to the anterior interosseous nerve (AIN), to reconstruct thumb and finger extension and flexion, respectively. Arrows indicate the sites of nerve coaptation.

Eight months after the injury, on the right side, through an axillary approach, the posterior division of the axillary nerve was transferred to the triceps long and upper medial head branches. The brachialis muscle was transferred to the flexor digitorum profundus and flexor pollicis longus employing a tendon graft harvested from the anterior tibialis muscle. Via a posterior forearm approach, nerves to the supinator were transferred to the posterior interosseous nerve (PIN). On the left side, using an anterior elbow approach, finger and thumb extension/flexion were reconstructed by transferring the nerves to the supinator to the PIN and the ECRB motor branch to the anterior interosseous nerve (Figure 1).

**Figure 2:**
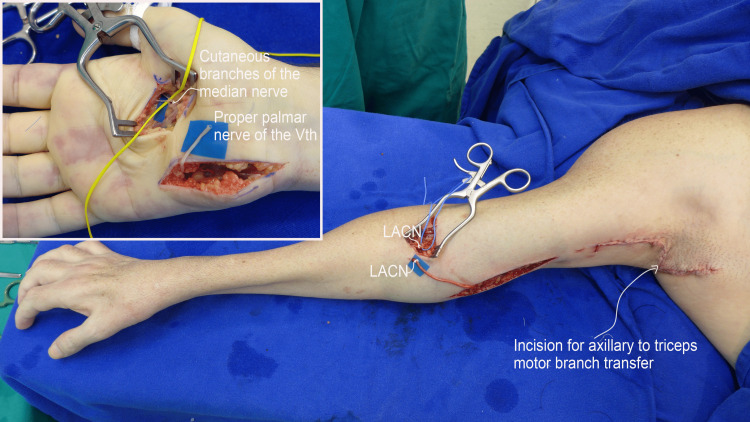
Intraoperative view of nerve transfers for sensory reconstruction. The lateral antebrachial cutaneous nerve (LACN) is transferred to the medial antebrachial cutaneous nerve. In the palm, sensory branches of the median nerve are connected to the medial proper palmar digital nerve of the little finger.

Bilaterally, for the sensory reconstruction of the medial border of the forearm and hand, the lateral antebrachial cutaneous nerve was transferred to the medial antebrachial cutaneous nerve (Figure 2), while cutaneous branches of the median nerve were transferred to the ulnar proper palmar digital nerve of the little finger in the palm [6-8].

**Figure 3:**
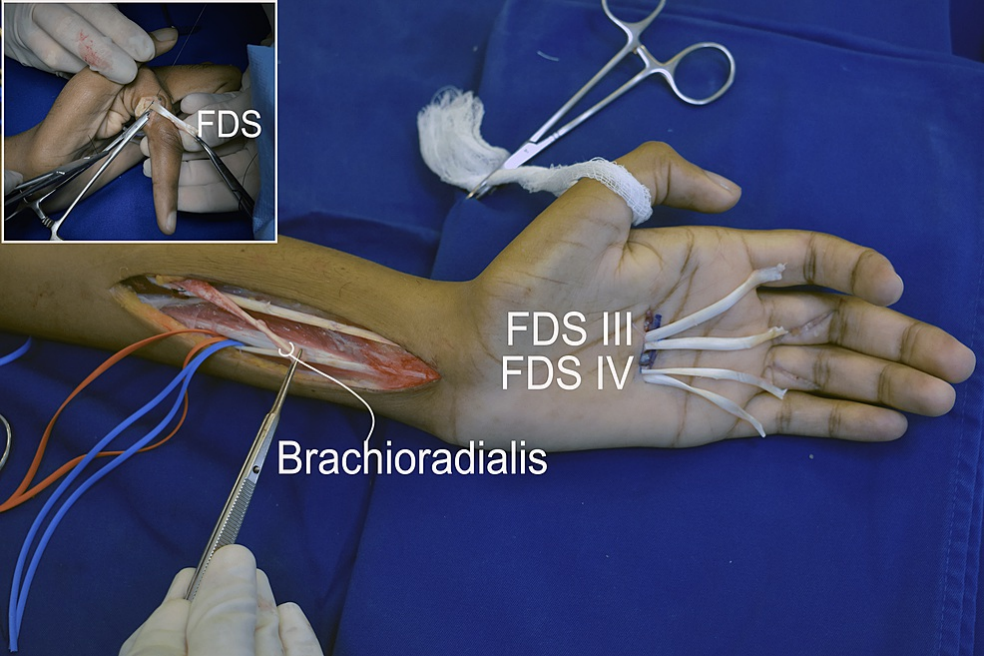
Intraoperative view of finger claw correction by transferring the flexor digitorum superficialis (FDS) of the third and fourth fingers to the extensor apparatus. Each FDS tendon was split into two slips. In the inset, the lateral slip of the FDS of the third finger is being sutured to the extensor tendon apparatus.

By 12 months after surgery, the patient had recovered elbow extension (M4) and finger flexion/extension on the right, scoring M4 and M3, respectively. On the left, the patient had recovered thumb and finger flexion/extension, all scoring M4 with a full range of motion. On both sides, the key pinch was impossible because of early flexion of the thumb and hyperextension of the index metacarpal-phalangeal joint (MP). We reoperated on the patient 24 months after the initial surgery to correct clawing bilaterally. We dissected the flexor digitorum superficialis (FDS) tendon of the middle and ring fingers at the proximal phalanx, retrieved them on the palm, and divided them into two slips each (Figure 3).

**Figure 4:**
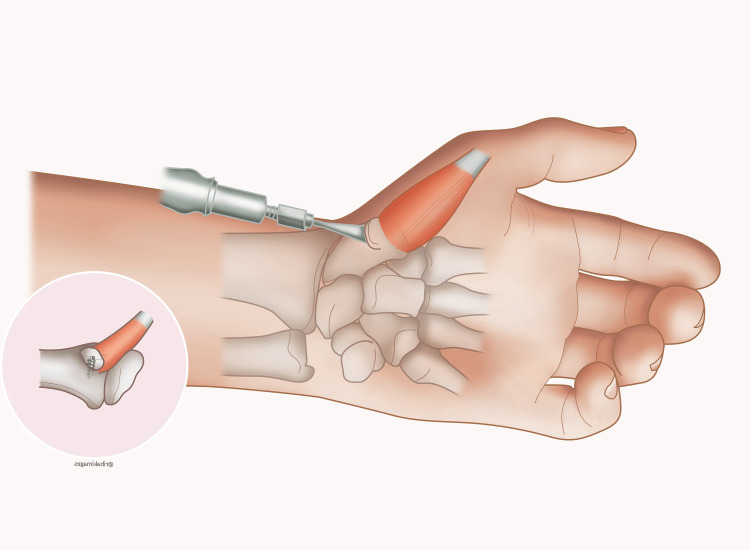
Schematic representation of bone-anchor fixation of the osteotomized scaphoid tubercle with attached muscle in the distal radius to improve thumb abduction. This art illustration is original. Authors own the copyrights.

The slips were passed along the radial side of the index, middle, and ring finger and sutured to the lateral band of the extensor tendon. In the little finger, the slip was passed around the ulnar side. For tension adjustment, the MP joints were flexed to 90º and the FDS pulled on half of its excursion before suturing. In the wrist, the brachioradialis tendon was attached to the FDS of the middle and ring fingers in half excursion and without FDS pulling. Nine months later, the key pinch was possible bilaterally. However, the patient requested pulp-to-pulp reconstruction between the thumb and index finger because he was unable to pick up small objects on flat surfaces like a table. During a new operation on the left hand, through a wrist volar approach extending to the thenar region, we dissected the thenar muscles and scaphoid tubercle, which was then osteotomized, maintaining thenar muscle attachments to it. The scaphoid tubercle was transferred proximally and fixed to the radius with a bone anchor, promoting thumb abduction (Figure 4).

Five years after nerve surgery, on the right side, elbow extension and finger flexion scored M4 and finger extension scored M3; MP extension was possible while the proximal interphalangeal joint (PIP) remained flexed. A full range of finger flexion and correction of the MP hyperextension was demonstrated. A key pinch was possible against the middle phalanx of the index finger. The patient complained about the lack of PIP extension, which hampered his bimanual activities. On the left side, thumb and finger flexion-extension scored M4 with a full range of motion. Clawing was absent. Pulp-to-pulp thumb and index pinch was restored, allowing the patient to grasp small objects placed on a table. Proximal advancement of the scaphoid tubercle had not stretched over time, maintaining the thumb in abduction. In Video 1, we show pre, intra, and five years postoperative views of our patient.

**Video 1:** In this video, we show pre-, intra-, and five years postoperative views of our patient. The surgical procedures are explained in detail. Watch here: https://youtu.be/529hMwSKCU8.

Light touch perception was restored on the distal ulnar surface of the forearm bilaterally, but not in the hand. Nociception was restored on the ulnar surface of the forearm and hand, but not on the fingertip of the little finger.

**Figure 5:**
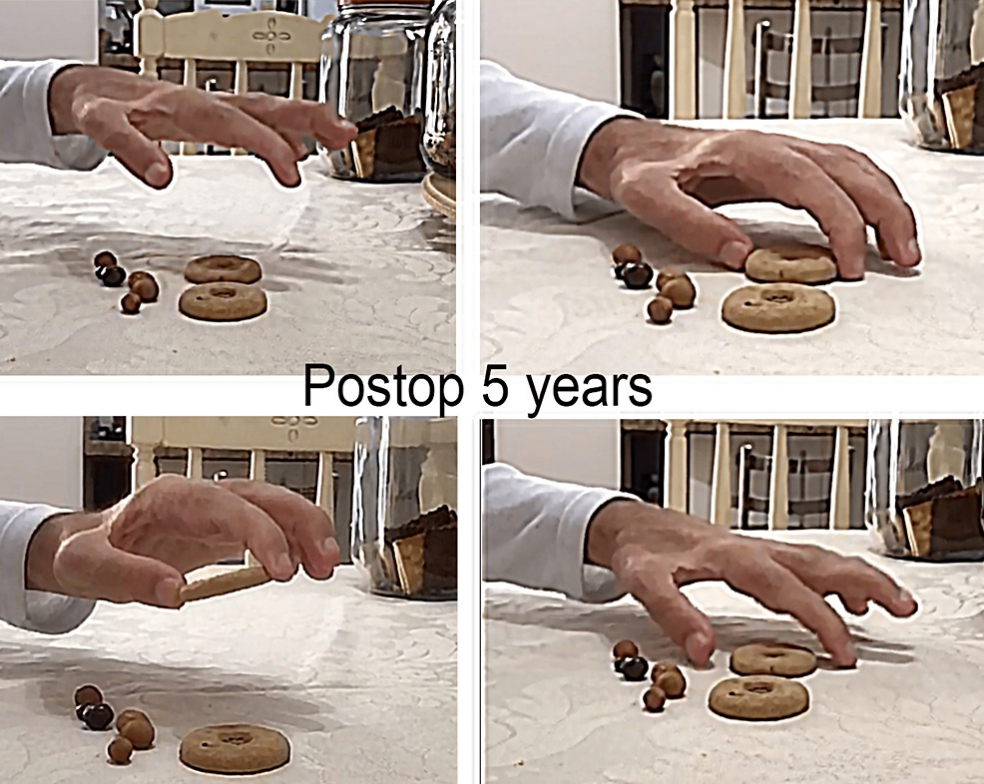
Five-year postoperative views. Preoperatively, no finger motion was possible. After surgery, note the patient's capacity to grasp a small object on a table.

Before the accident, the patient was right-handed, but he became left-handed after surgery. Using his left hand, he was able to grasp both large and small objects lying on a table (Figure 5).

He used his right hand to assist with bimanual activities and to receive objects transferred from the left hand, such as a smartphone. The patient used his right hand independently only as a hook to pull on the break of his wheelchair.

In Table 1, we summarize the results of surgical reconstruction. In Table 2, we indicate the patient’s functional improvement, based on the Tetraplegic Upper Limb Activities Questionnaire [9], which clearly indicates not only improvements from the baseline but also the transfer of handedness to his left hand.

**Table 1:** Postoperative results for motion reconstruction. Elbow and wrist extensions were measured using a push-and-pull dynamometer (Baseline®, New York, USA). Grasping was measured with the Jamar dynamometer (Baseline®), whereas pinch strength was assessed with a pinch-gauge (Baseline®). Finger and thumb flexion/extension strength was scored according to the British Medical Research scale. The thumb span was measured using a ruler as the distance between the thumb pulp to the pulp of the index finger in maximal active hand opening. Sensory evaluation was carried out using the 2 gr monofilament (Sorry, São Paulo, Brazil). Nociception was tested using an eyebrow tweezer.

Assessment	60 months postoperative	
	Right	Left
Wrist extension strength (kg)	9	9.5
Grasping (kg)	5.5	2.5
Key pinch (kg)	0.5	0.5
Pulp-pinch (kg)	Not possible	0.3
Tripod-pinch (kg)	Not possible	Not possible
Elbow extension strength (kg)	4	4
Shoulder abduction (º)	170, M4	170, M4
Shoulder external rotation (º)	120, M4	120, M4
Finger extension strength	M3	M4
Thumb extension strength	M3	M4
Finger flexion and flexor pollicis longus (FPL) strength	M4	M4
FPL strength	M4	M4
Thumb span (cm)	4	9

**Table 2:** Performance in daily activities according to the Tetraplegic Upper Activities Questionnaire 60 months after nerve surgery. Preoperatively, the scores were 10 and 10. For all activities, our patient favored the use of the left hand.

Activity (best hand)	Performance (1-5)	Satisfaction (1-5)	Aids used
Writing with a pen	1	1	Yes
Handle banknotes and credit cards in/out of the wallet	1	1	No
Pick up items from a flat surface without sliding off the edge	3	4	No
Grasp and re-position book/tablet	2	2	No
Eating with a fork/spoon	4	4	Yes
Cut food when eating	1	1	No
Drinking from a bottle	4	5	No
Shaving/putting on make-up	1	1	No
Adjust upper half clothing pulling down back to waist level	1	1	No
Open (previously opened) bottles and jars using your hands	1	1	No
Total raw score	19/40	21/40	

## Discussion

In our patient’s right hand, our nerve transfer surgery was successful at reconstructing elbow extension and both thumb and finger extension, confirming previous findings [2,10]. On the right side, despite extensor digitorum reinnervation using the nerve to the supinator and our attempt to correct clawing, a full proximal interphalangeal extension was not possible. This likely occurred not only from the elongation of our FDS tendon transfer, powered by the brachioradialis muscle, but also from the elongation of the extensor tendon central slip. One possible approach to correct this deformity is to shift the attachment of our tendon transfer from the lateral to the central band of the extensor tendon, aiming to enhance proximal interphalangeal extension. We refrain from utilizing static procedures to correct clawing, as they constrain metacarpophalangeal extension [11]. The patient is now using PIP extension orthotics to improve bimanual hand function. Hand opening is now improved with the hand in supination and the wrist supported, to avoid wrist extension. This is caused by the absence of wrist flexors, in which situation the activation of finger extensors drives the wrist into extension, hampering hand opening. However, many patients with tetraplegia who have had their nerve to the supinator transferred to the PIN can overcome the absence of wrist flexors by using gravity to flex their wrist [10].

On the right side, for thumb and finger flexion reconstruction, we opted to transfer the brachialis muscle rather than the nerve to the ECRB, because wrist extension was weak. We observed a reduced thumb span in the right versus left land, which probably reflects using the weaker nerve to the supinator as the donor for PIN reinnervation. The strength of elbow extension reconstruction by nerve transfer matched that of left-side spontaneous recovery (i.e., 4 kg). For comparison, the biceps-to-triceps muscle transfer yielded an average of 2.9 kg of elbow extension strength [12].

On the left side, finger flexion and extension were reconstructed using nerve transfers only. After surgery, despite the left hand being weaker, the patient became left-handed. Reasons for this likely include superior hand opening, finer finger flexion control, and thumb positioning, relative to the right hand, thereby allowing a pulp-to-pulp pinch with the index finger. It is possible that, rather than strength, superior fine motor control prompted this patient’s left-hand preference.

Key pinch reconstruction has been uniformly recommended for tetraplegic hands at the expense of opposition [4]. However, in normal individuals, key pinch is only used 9% of the time during routine daily activities, as opposed to pulp-to-pulp pinch, which is used 38% of the time [11]. It is possible that patients with tetraplegia have greater demand for pulp-to-pulp pinch, so they may grasp small objects on a table, such as food. In individuals without upper extremity neurological deficits, the time required for them to feed themselves involves roughly one hour of hand use per day. Pulp-to-pulp pinching is used for approximately 56 minutes, with cylindrical grasping used for about four minutes (e.g., drinking water from the bottle) and lateral pinch only for roughly 4:30 minutes [13].

To create a pulp-to-pulp pinch, we reanimate finger flexors and extensors via nerve transfers, corrected clawing using tendon transfers, and repositioned the thumb using proximal advancement of the scaphoid tubercle with the thenar muscles attached. Others have proposed the first carpometacarpal arthrodesis for better positioning of the thumb [4]. However, this clearly limits hand span.

## Conclusions

In cases of cervical spinal cord injury, upper limb surgery can enhance hand function, promoting greater independence in daily tasks. When donor nerves are viable for reanimating thumb and finger motion, procedures like pulp-to-pulp pinch reconstruction can reinstate fine motor control. This enables patients to delicately manipulate small objects across a flat surface with heightened precision.
